# Interaction of Near-Infrared (NIR)-Light Responsive Probes with Lipid Membranes: A Combined Simulation and Experimental Study

**DOI:** 10.3390/pharmaceutics15071853

**Published:** 2023-06-30

**Authors:** Hugo A. L. Filipe, André F. Moreira, Sónia P. Miguel, Maximiano P. Ribeiro, Paula Coutinho

**Affiliations:** 1 CPIRN-IPG—Center of Potential and Innovation of Natural Resources, Polytechnic Institute of Guarda, 6300-559 Guarda, Portugal; spmiguel@ipg.pt (S.P.M.); mribeiro@ipg.pt (M.P.R.); coutinho@ipg.pt (P.C.); 2 Coimbra Chemistry Center, Institute of Molecular Sciences (CQC-IMS), University of Coimbra, 3004-535 Coimbra, Portugal; 3 CICS-UBI—Centro de Investigação em Ciências da Saúde, Universidade da Beira Interior, 6200-506 Covilhã, Portugal

**Keywords:** near-infrared (NIR)-light responsive probes, lipid membranes, molecular dynamics simulations, photothermal therapy (PTT), photodynamic therapy (PDT)

## Abstract

Cancer is considered a major societal challenge for the next decade worldwide. Developing strategies for simultaneous diagnosis and treatment has been considered a promising tool for fighting cancer. For this, the development of nanomaterials incorporating prototypic near-infrared (NIR)-light responsive probes, such as heptamethine cyanines, has been showing very promising results. The heptamethine cyanine-incorporating nanomaterials can be used for a tumor’s visualization and, upon interaction with NIR light, can also produce a photothermal/photodynamic effect with a high spatio-temporal resolution and minimal side effects, leading to an improved therapeutic outcome. In this work, we studied the interaction of 12 NIR-light responsive probes with lipid membrane models by molecular dynamics simulations. We performed a detailed characterization of the location, orientation, and local perturbation effects of these molecules on the lipid bilayer. Based on this information, the probes were divided into two groups, predicting a lower and higher perturbation of the lipid bilayer. From each group, one molecule was selected for testing in a membrane leakage assay. The experimental data validate the hypothesis that molecules with charged substituents, which function as two polar anchors for the aqueous phase while spanning the membrane thickness, are more likely to disturb the membrane by the formation of defects and pores, increasing the membrane leakage. The obtained results are expected to contribute to the selection of the most suitable molecules for the desired application or eventually guiding the design of probe modifications for achieving an optimal interaction with tumor cell membranes.

## 1. Introduction

The worldwide incidence and mortality of cancer are well documented, with millions of new cases and deaths registered every year [1]. Different types of cancer can also require different therapeutic approaches, such as chemotherapy, radiotherapy, and hyperthermia. In conventional hyperthermia, the whole body or body regions are treated by creating temperature gradients from the surface to the interior regions of the body [2]. In turn, localized hyperthermia promotes a tissue-specific temperature increase, which is commonly achieved by using focused ultra-sounds or infrared (NIR) radiation in combination with potentially activated molecules or materials [3]. Particularly, the utilization of photothermal therapy (PTT) is dependent on several factors such as the laser parameters, the light-to-heat conversion efficiency of the probes, and the tumor accumulation of the photosensitive molecules [4,5]. Moreover, the light-triggered approaches can also be explored to mediate a photodynamic effect, i.e., create radical oxygen species in response to the light exposure, using drugs as intermediaries, known as photosensitizers or photosensitizing agents. This response can be activated by various light sources, such as a laser or LEDs, and used as a localized treatment, treating specific parts of the body (e.g., killing cancer and other abnormal cells) [6,7].

Typical molecules used in phototheragnostic modalities are the heptamethine cyanine dyes. These molecules are used in a dual activity mode, having the possibility to combine PTT and photodynamic therapy (PDT) [5,8,9,10]. Another valuable application of this type of molecules is in the context of bioimaging techniques [11,12,13,14]. Usually, these molecules have low solubility, low tumor uptake, high blood clearance, and acute toxicity [5]. A common strategy for overcoming these problems is the incorporation of the active molecules in nanoparticles or nanocarriers, resulting in higher solubility, blood circulation, tumor accumulation, residence time in the tumor, and biocompatibility and providing NIR absorption to the particles [15,16]. Once these molecules are delivered to the target tissue, cancer cells may be eliminated upon irradiation with an NIR laser [5,17,18,19].

**Figure 1:**
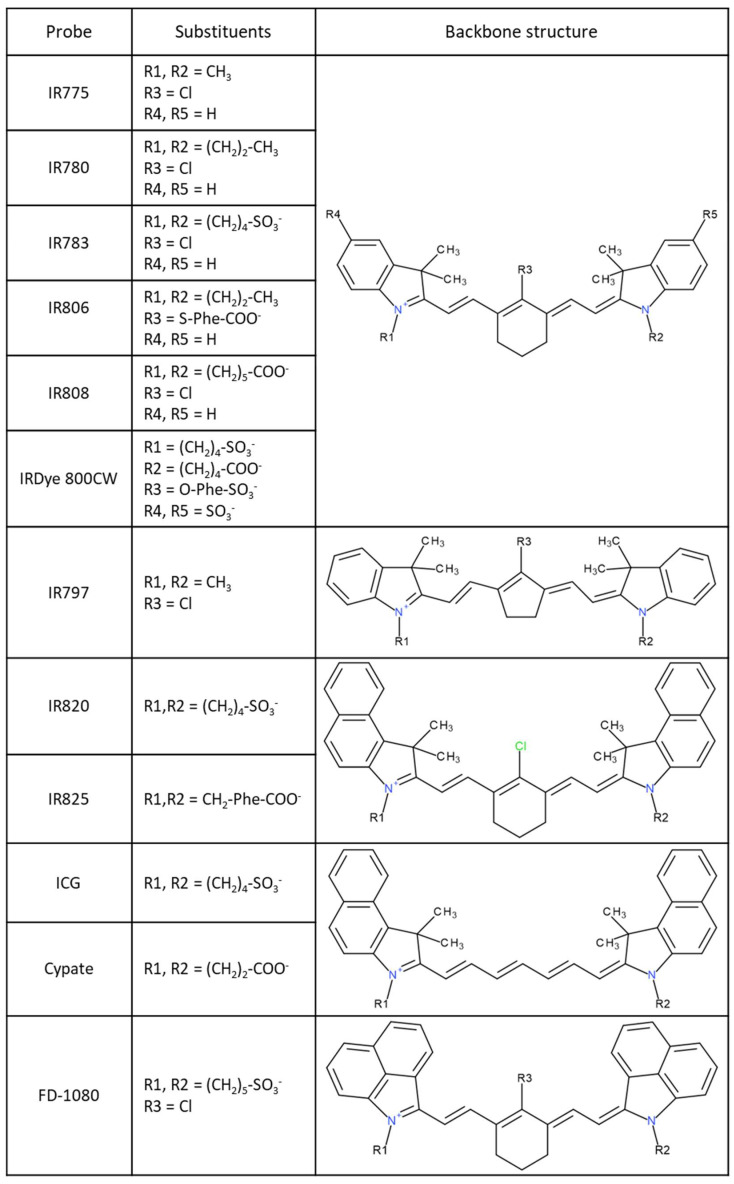
Schematic representation of the 12 NIR probes studied in this work. Different probes are grouped according to their backbone structural similarity.

The application of NIR molecules in biomedicine has increased in recent years; several options are available commercially. In this work, we have focused on the behavior of 12 different probes (structures are resumed in Figure 1), recently reviewed elsewhere [11]. These molecules may be divided into five typical backbone structures, or typical structures, with the heptamethine cyanine spacer connecting two heteroring (indole) systems. In the first typical structure, Structure I, there are IR775, IR780, IR783, IR806, IR808, and IRDye 800CW. Herein, one of the most explored probes is IR780, where R1 and R2 are neutral. Moreover, most of the probes have a chlorine atom as an R3 substituent, but other chemical groups may also be present, such as in the case of IR806 and IRDye 800CW. For R4 and R5 substituents, they are usually hydrogens, with the exception of IRDye 800CW, which presents SO_3_^−^. In Structure II, i.e., IR797, the main backbone structure includes a middle five-member ring group, instead of a six-member ring. In Structures III (IR820 and IR825), IV (ICG and Cypate), and V (FD-1080), the interior region of the structure may be different, but all of them present three rings on hetero-ring systems. For a typical Structure IV, the center ring system is absent. In turn, the typical Structure V differs from that of type III by the organization of the two hetero-ring systems on the molecular edges. The photophysical properties of these molecules and their biological applicability have also been reviewed [11]. All five of the different structures are generally associated with high molar absortivities, with FD-1080 presenting the capacity to interact with light at larger wavelengths than for the other probes. Moreover, different works in the literature already tested the applicability of IR780 [20,21,22,23], IR783 [24,25], IR808 [26,27], and IR820 [28,29] in tumor therapy, whereas the IRDye 800CW [30,31] was already the subject of clinical trials (ClinicalTrials.gov Identifier: NCT02736578, accessed on 24 March 2023).

However, despite the information available, the selection of the right molecule for a given application remains a big challenge. In this context, the establishment of structure–activity relationships has an added value to the performance prediction of the different heptamethine cyanine molecules. Therefore, the systematic characterization of the NIR probes’ interaction with model membranes can provide important knowledge, since the plasma membrane is a ubiquitous barrier for cell permeation and can also be a therapeutic target [32]. Overall, it is expected that better performances correlate with the interaction of the molecules with lipid cell membranes. This molecule–cell membrane interaction, as well as the intrinsic ability of the molecules to perturb the membranes of the cancer cells, is expected to depend on the balance between neutral or charged molecular substituents, present on the NIR probes, and the composition of the heteroring systems. 

In this work, we characterized the interaction of different NIR-light responsive probes with lipid membrane models by molecular dynamics (MD) simulations to identify the molecules with a higher intrinsic ability to perturb lipid membranes. Then, the insights resulting from the simulations were validated experimentally, using lipid membrane leakage assays. The replication of this type of work for other molecules in different lipid membranes can be used to complement the information about the biological properties of the NIR probes with their behavior when inserted in lipid bilayers for a better performance on the treatment of cancer. As a final goal, we aim to contribute to the selection of the best NIR probes for a desired application or, eventually, to guide the probe modifications for achieving an optimal interaction with the membranes of the tumor cells.

## 2. Materials and Methods

### 2.1. All-Atom MD Simulation

MD simulations and analysis were carried out with GROMACS version 2021 [33]. The CHARMM36 force field was employed for lipids [34], together with the updated version of the original Beglov and Roux parameters [35] for the ions [36], the CGenFF v4.6 for the NIR molecules [37], and the modified version of the TIP3P model [38,39] to be used with the CHARMM force field for the water molecules [40]. The parametrizations of NIR probes were carried out using the Ligand Reader & Modeler tool [41] from the CHARMM-GUI platform [42]. The pure membrane systems were built using the Membrane Builder tool from CHARMM-GUI [43]. Lipid bilayers composed of 1-palmitoyl, 2-oleoyl-*sn*-glycero-3-phosphocholine (POPC) were used, as this lipid is the most abundant lipid in cell membranes [44] and is therefore conventionally used in this type of simulation [45,46]. For the pure membrane system, a total of 256 lipids and 50 water molecules/lipid were added. The membrane systems were equilibrated with the CHARMM-GUI protocol, including a minimization step and several small NVT and NPT equilibration steps with position restraints that were gradually alleviated until the restraint-free production run [47]. All the topologies files obtained were converted to be compatible with the GROMACS software by the CHARMM-GUI platform [47]. The production run for the POPC membrane was extended until 200 ns.

The construction of the systems containing the NIR probes was carried out by placing four NIR molecules positioned in the center of the lipid membrane. The systems were subsequently neutralized with sodium (Na^+^) or chloride (Cl^−^) ions, depending on the system. No further ions were added to the systems. All systems were minimized, followed by NVT and NPT 100 ps equilibration runs with a 1 fs integration step. The production runs were carried out for 200 ns. Three replicates were run for each system, changing the initial position of the NIR molecules and with the initial velocities of the particles randomly attributed.

All production runs were performed at NPT conditions, with simulations settings similar to those of a previous work [48]. An integration step of 2 fs was employed. Periodic boundary conditions were always applied. The electrostatic interactions were modeled with the particle-mesh Ewald (PME) method [49], with a 1.2 nm cut-off. A force-based switch function with a 1.0–1.2 nm range was applied for the van der Waals interactions cut-off. The NIR molecules together with the membrane lipids and the water molecules together with ions were independently coupled to temperature baths at 298.15 K, using the Nose–Hoover algorithm [50,51] and with a time constant for coupling (tau-t) of 1 ps. The pressure was maintained constant at 1 bar with a barostat employing the Parrinello–Rahman algorithm [52] with a semi-isotropic scheme, a coupling constant of 5 ps, and a compressibility of 4.5 × 10^−5^ bar^−1^. Constraints in the H-bonds were applied using the LINCS algorithm [53], and no dispersion corrections were used.

The visualization of the trajectories was carried out with Visual Molecular Dynamics (VMD) v.1.9.3 [54]. The analysis of the location, orientation, area/lipid, and hydrogen bonds was performed with standard GROMACS tools. The first 50 ns of each run were discarded from the analysis.

### 2.2. Experimental Measurement of the CBF Release from Lipid Vesicles

#### 2.2.1. Preparation of CBF-Loaded Red Blood Cell (RBC)-Membrane-Derived Vesicles

The preparation of red blood cell (RBC)-membrane-derived vesicles was performed by adapting a method previously described in the literature [55]. Briefly, EDTA-stabilized blood was obtained from adult mice and centrifuged at 500× *g* for 5 min at 4 °C to recover the RBCs. The pellet was rinsed three times with cold NaCl (150 mM). The RBCs’ hemolysis was then promoted via incubation in ice-cold phosphate-buffered saline (PBS) solution 0.25× for 20 min. Afterwards, the sample was centrifuged at 800× *g* for 5 min, and the resulting pellet was recovered and washed two times with PBS 1×. The RBC-membrane-derived vesicles were then formed by sonication for 10 min and extrusion through a 400 nm polycarbonate porous membrane using an Avanti mini extruder (Avanti Polar Lipids). The 5(6)-Carboxyfluorescein (CBF)-loaded RBC-membrane-derived vesicles were then prepared by mixing 10 mL of the vesicles’ solution (0.4 mg/mL) with 1 mL of CBF (1 mM). The mixture was sonicated for 10 min and extruded nine times through a 200 nm polycarbonate porous membrane using an Avanti mini extruder (Avanti Polar Lipids). The resulting CBF@RBC-membrane-derived vesicles were centrifuged, and the non-loaded CBF was removed. The CBF@RBC-membrane-derived vesicles were also washed with PBS 1x using a Vivaspin tube (Mw cut-off: 10,000 Da) to remove CBF molecules adsorbed at the vesicles’ surface. The supernatant was stored for quantification purposes. The size and charge of the produced membrane-derived vesicles were characterized in Zetasizer Nano ZS equipment (Malvern Instruments, Worcestershire, UK). The measurements were performed in ultrapure water at 25 °C in a disposable capillary cell. Furthermore, the amount of CBF loaded in the RBC-membrane-derived vesicles was determined by measuring the supernatant fluorescence in a spectrofluorometer (Spectramax Gemini XS, Molecular Devices LLC, San Jose, CA, USA) at an excitation/emission wavelength of λex = 492 nm and λem = 517 nm and using a calibration curve (Flu = 1 × 10^7^ C + 1567.7; r² = 0.9944). The encapsulation efficiency of the CBF@RBC-membrane-derived vesicles was calculated using Equation (1):

(1) $$Encapsulation\;efficiency\;\left(\%\right)=\frac{\left(Initial\;CBF\;weight\;–\;CBF\;weight\;in\;supernatant\right)}{Initial\;CBF\;weight}\times 100$$

#### 2.2.2. Characterization of the CBF Release in the Presence of IR780 and IR820

The CBF release from the CBF@RBC-membrane-derived vesicles was analyzed in the presence of increasing concentrations of IR780 or IR820. Briefly, 400 µL of CBF-loaded vesicles (final mass concentration of 320 µg/mL) was mixed with IR780 or IR820 solution (10% *v*/*v* methanol in PBS), with a final IR concentration of 0, 2.5, 5, 10, and 20 µM (final methanol concentration of 2% *v*/*v*), followed by incubation at room temperature for 4 h. At different time points, the sample was recovered to a Vivaspin tube (Mw cut-off: 10,000 Da) and centrifuged. The obtained filtrate was then analyzed in a spectrofluorometer (Spectramax Gemini XS, Molecular Devices LLC, San Jose, CA, USA) (λex = 492 nm and λem = 517 nm) to determine the amount of CBF released. CBF@RBC-membrane-derived vesicles non-exposed to IR molecules were used as the control. The data are presented as the mean ± standard deviation (s.d.) (*n* = 3). 

## 3. Results and Discussion

### 3.1. Detailed Characterization by MD Simulations

**Figure 2:**
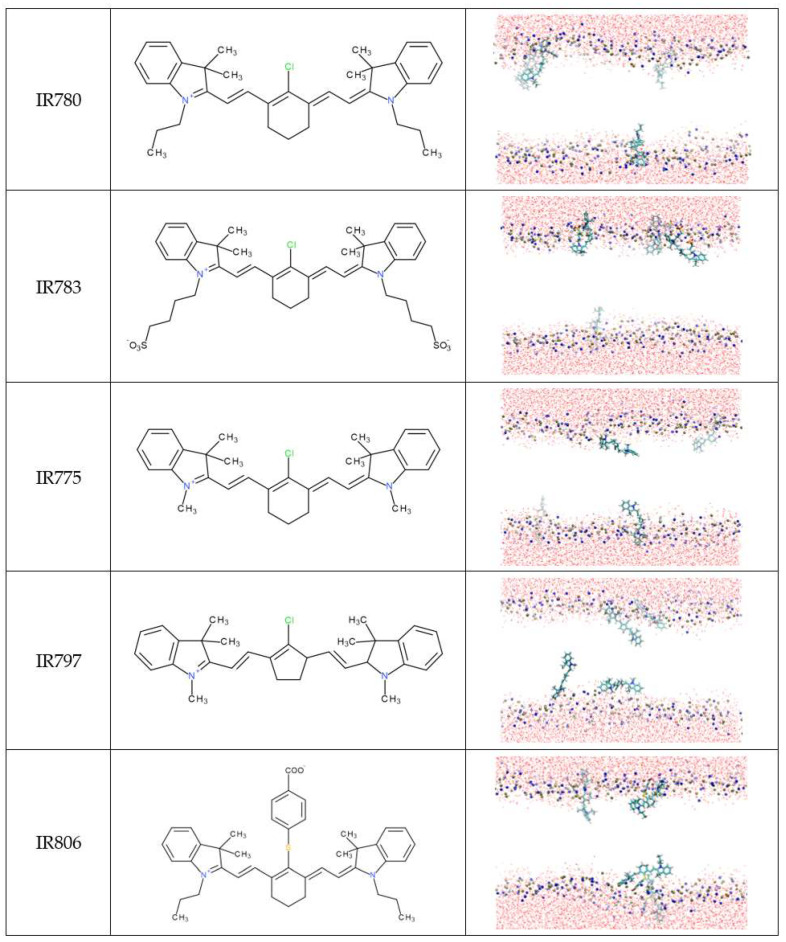
Final snapshots of representative molecular dynamics simulations for each of the NIR probes included in Group 1, according to their preferential location and orientation on the lipid membrane. The structure of each molecule is also shown for an easier interpretation of the location and orientation of the different molecules. Atom colors are: C (cyan), O (red), P (orange), N (blue), S (yellow), Cl (green), and H (white). Membrane lipids are omitted for clarity, with the exception of the P and N atoms.

**Figure 3:**
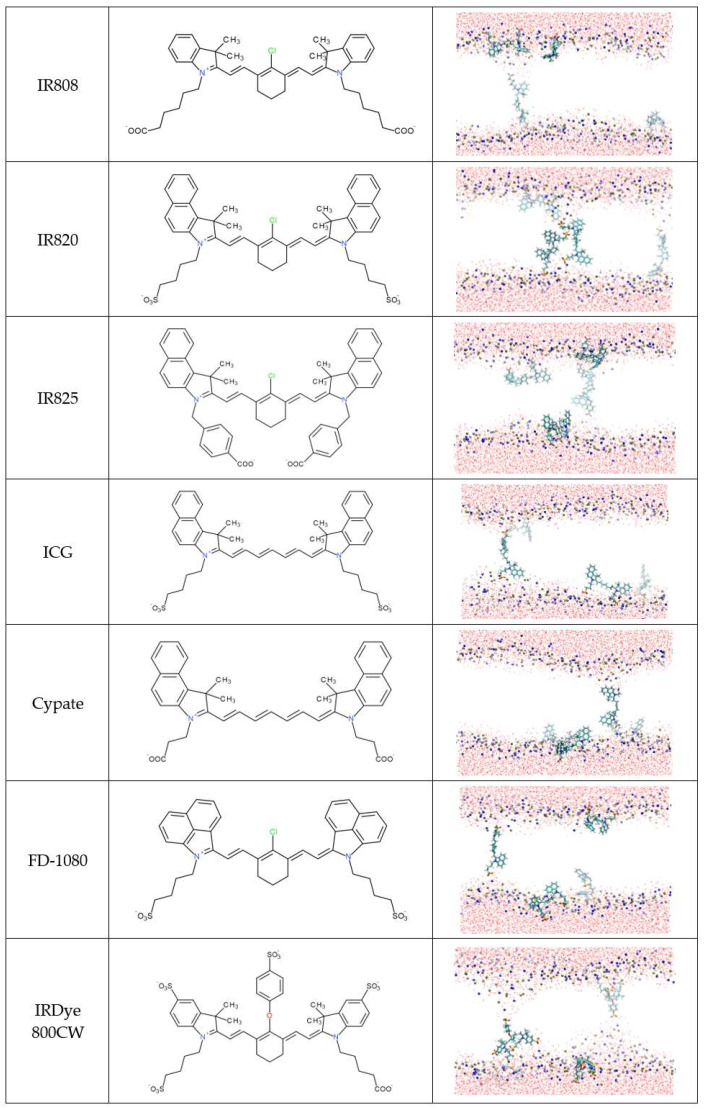
Final snapshots of representative molecular dynamics simulations for each of the NIR probes included in Group 2, according to their preferential location and orientation on the lipid membrane. The structure of each molecule is also shown for an easier interpretation of the location and orientation of the different molecules. Atom colors are: C (cyan), O (red), P (orange), N (blue), S (yellow), Cl (green), and H (white). Membrane lipids are omitted for clarity, with the exception of the P and N atoms.

MD simulations were used to characterize, in detail, the interaction of the NIR probes with the lipid bilayers. Figure 2 and Figure 3 show the final snapshots of the 200 ns simulations. 

Considering the final location of the probes in the membrane, the 12 NIR probes were divided into two groups. Group 1 (Figure 2) is composed of molecules that are preferentially located at the membrane/water interface in all simulations. In general, these molecules have neutral R1 and R2 substituents. The exception is IR783, where these substituents are charged. The molecule IR806 is also distinct from the others, since it has a single charged group at the R3 substituent.

Group 2 (Figure 3) comprises the remaining seven molecules, which presented a different behavior. During their interaction with the lipid membrane, all molecules from group 2 end up forming defects or even creating pores on the lipid bilayer, which may suggest a higher propensity for inducing perturbations in the membranes of the cells. All of these molecules have in common the presence of charged R1 and R2 substituents that may help the molecule to span the whole membrane thickness while deeply inserted in the membrane.

Additional notes should be considered regarding the group attribution of specific molecules. Despite presenting charged R1 and R2 substituents, the IR783 probe was attributed to group 1. To explain this observation, we may speculate that it is because most of the molecules of group 2 have three rings on the heteroring systems that may pull down the molecules deeper in the membrane. The exception to this structural motif on group 2 molecules is IR808, which, similar to IR783, has only two ring systems. However, in IR808, the R1 and R2 groups are one carbon longer, which may help the molecule to span the whole membrane thickness. Another singular molecule in group 2 is the IRDye 800CW that has a very complex structure, where, once inserted in the membrane, the charged groups may pull down the lipids, creating larger membrane deformations, as shown in Figure 3, Figures S1 and S2. The induction of membrane defects and, eventually, membrane pores is a molecular mechanism described for the activity of a large variety of molecules [56,57,58,59]. From several reports available in the literature, we highlight the ability of larger kaempferol derivatives to span the whole membrane thickness, similar to the molecules categorized in group 2, and induce the formation of pores in lipid membranes [58]. 

#### 3.1.1. Location of the NIR Probes on the Lipid Membrane

**Figure 4:**
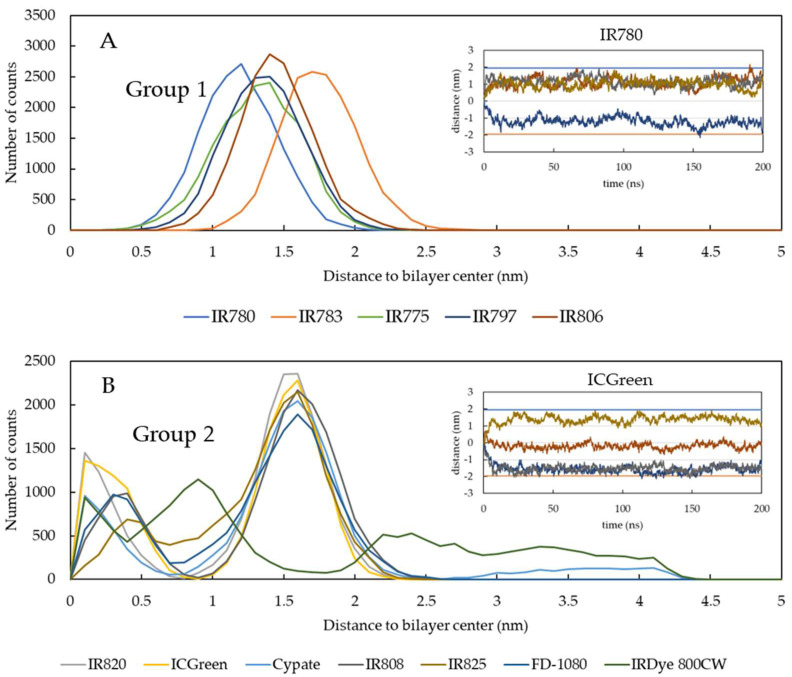
Analysis of the NIR probes’ location on the lipid membrane, determined by the distribution of the number of counts of the COM as a function of the distance to the bilayer center. Plots (**A**,**B**) show the position distributions for the molecules from group 1 and group 2, respectively. The insets on plots A and B show the time dependence of the positions for IR783 and ICGreen, respectively.

We analyzed the time dependence of the location of the molecules’ center of mass (COM) on the membrane and created histograms to quantitatively describe the location of the probes in a particular position on the lipid membrane thickness. The count histograms distribution is the direct result from the regions sampled by the different molecules in each simulation. The results are shown in Figure 4A for the molecules from group 1 and in Figure 4B for the molecules from group 2. As shown in the inset plots of Figure 4, the molecules initially inserted in the center of the membrane changed their position until they reached a preferential location. 

The molecules from group 1 have a single distribution region on the membrane interface. However, despite all molecules being located preferentially in the same region, some locate deeper than others. Molecules with neutral R1 and R2 groups, such as IR780, present a preferential location deeper in the membrane. In fact, IR780 is likely to be the most hydrophobic molecule in the study, as it only carries the charge associated with the charged nitrogen of the ring systems. Moreover, IR780 has no charged substituents, since the two substituents on R1 and R2 are propyl groups. On the other hand, IR783, a molecule with charged R1 and R2 groups, presents a shallower location in the membrane. In turn, molecules from group 2, since their structure has several charged groups, also locate preferentially at the membrane/water interface. This is a common behavior of charged molecules interacting with lipid membranes [60]. To the best of our knowledge, the interaction of the molecules addressed in this work with lipid bilayers has never been reported. However, the interaction of related carbocyanine membrane probes with lipid bilayers has been characterized [61,62,63,64]. Depending on the full structure of the probe, the carbocyanine backbone has been reported to be located between 1.0 and 1.5 nm from the bilayer center [62,64]. Additionally, molecules with a larger polymethine bridge (seven carbons for the molecules in our work) have been reported to locate deeper in the membranes [64]. Overall, the preferential location for the COM of the molecules in our simulations is in agreement with that reported for carbocyanine membrane probes. However, the presence of R1 and R2 charged substituents, namely, on group 2 molecules, is responsible for the shallower location in the membrane structure. However, contrary to the molecules from group 1, some of the group 2 molecules are preferentially located deeper in the membrane during the simulation time, as shown in the inset of Figure 4B, those being responsible for driving the formation of defects in the membrane (Figure 3). 

This immediate comparison of the distribution of the location of the molecules on the lipid bilayer gives insights into the possible perturbations that these molecules may create on cell membranes during a therapeutic application. Molecules from group 2, constituted by charged R1 and R2 groups, should be less hydrophobic than the molecules from group 1, without charged groups. However, due to the deeper location identified on some of the group 2 molecules, these are expected to induce a higher perturbation of lipid membranes and, consequently, are predicted to be more efficient in the induction of membrane leakage that will ultimately lead to a higher activity towards cell death.

#### 3.1.2. Orientation of the NIR Probes on the Lipid Membrane

**Figure 5:**
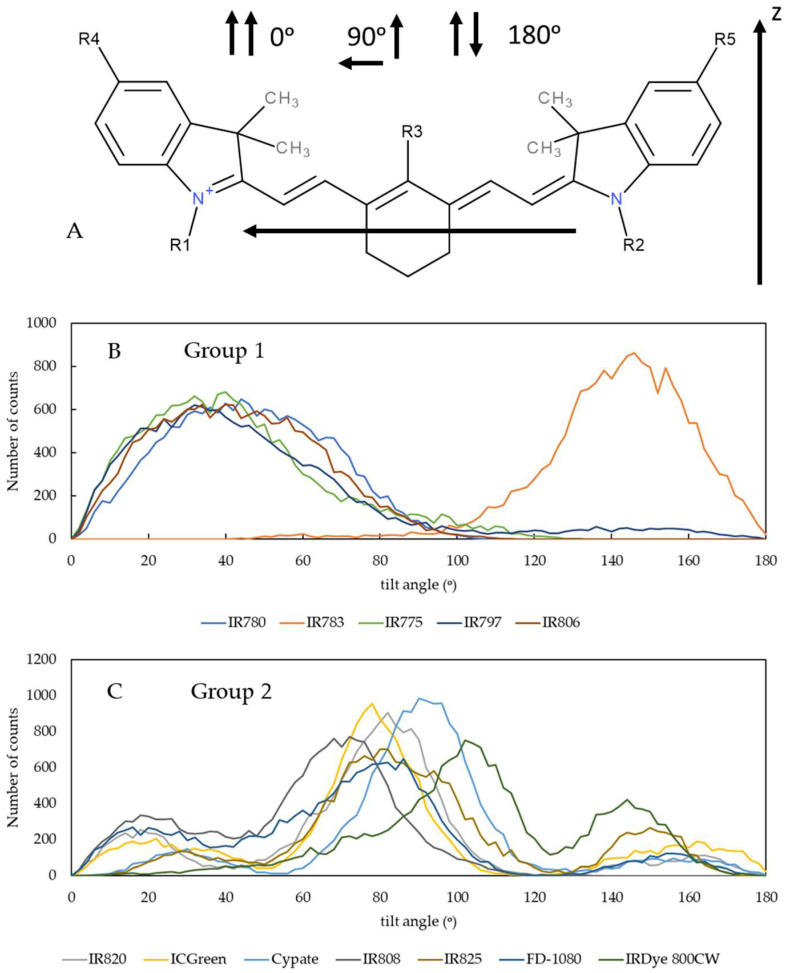
Orientation of the NIR probes on the lipid membrane, determined by the distribution of the number of counts for the angle between the z-axis perpendicular to the membrane surface and the vector defined as the long axis of the NIR molecules. Panel (**A**) shows the definition of the angles, where 90° means that the molecule’s long axis is parallel to the membrane surface and 0° or 180° mean that the long axis is perpendicular to the membrane surface. Plots (**B**,**C**) show the angle distributions for the molecules from group 1 and group 2, respectively.

The characterization of the orientation of drug-like molecules, such as the NIR probes simulated in this work, is very important to predicting their behavior during the interaction with the lipid membranes [45,46,65,66,67,68,69]. Therefore, the orientation of the molecules on the lipid membrane was analyzed from the distribution of the tilt angle formed by the long axis of the NIR molecules and the vector normal to the membrane surface, i.e., the *z*-axis on the simulation box, as shown in Figure 5A. Other examples of a similar analysis have been reported for carbocyanine probes [70]. The horizontal orientation on the membrane surface corresponds to a 90° tilt angle. If the vector defined in the molecule aligns with the *z*-axis, the orientation has an angle of 0°, and if the vector defined in the molecule aligns opposite to the *z*-axis, the orientation has an angle of 180°.

The analysis of the orientation of the molecules reveals that molecules from group 1 prefer to be inserted with a vertical orientation in the lipid membrane, with a maximum angle distribution around 30–50°. They do not orient completely vertically, as this would correspond to an angle of 0° or 180°. It has been reported in the literature that, dependent on the lipid composition, the orientation angle of carbocyanine probes is found to deviate clearly from 90°, although this is the general preference [70]. The preferential angle distribution around 30–50° found in our study may be justified by the larger size of the heptamethine bridge that favors the vertical packing of the group 1 molecules within the lipid chains. Nevertheless, although qualitatively similar, the orientation of the IR783 is inverted compared to the orientation of the other Group 1 probes, meaning that this molecule prefers an inverted vertical orientation. We may speculate that this distinct behavior should be related to the more external location of IR783 in the membrane, with a charged substituent facing the water. 

As anticipated from the analysis of Figure 2 and Figure 3, the orientation of the molecules from Group 2 shows a more complex behavior. Here, the molecules located at the membrane/water interface have a horizontal orientation, corresponding to angles around 90º. On the other hand, the molecules that deeply insert into the membrane acquire a vertical orientation, both with low and high tilt angles, depending on the specific orientation of the long-axis vector of the molecules. For these deeper-located molecules, the vertical orientation also favors their packing within the lipid chains. The conjugation of these three types of orientations originates from the complex histograms shown in Figure 5B. From the analysis of Figure 5A,B, it is possible to observe that molecules from group 2 that orientate vertically have larger angle values, i.e., more towards 0° and 180° than molecules from group 1. This may be understood as being due to the deeper insertion of these group 2 molecules in the lipid membrane and the need to accommodate them between the lipid tails.

#### 3.1.3. Increase in the Membrane Surface

**Figure 6:**
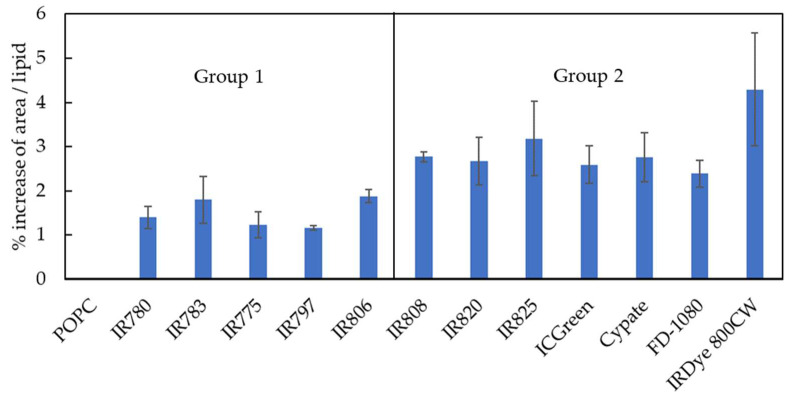
Membrane expansion determined as the increase in the area per lipid due to the presence of the different NIR probes in the lipid membrane. Pure POPC was used as a reference, being represented with a 0% increase.

As an additional form of characterizing the membrane perturbation induced by the NIR probes, we analyzed the increase in the membrane surface by calculating the area per lipid upon the insertion of the NIR molecules on the membrane. The area per lipid is used as an important analysis for the validation of the simulation of lipid membranes and is a measure of the average area that a molecule occupies in the membrane surface [45,65]. In this case, an increase in the area per lipid should be correlated with an expansion of the membrane due to the formation of membrane defects when interacting with NIR molecules. The results are shown in Figure 6. For the five molecules from Group 1, it may be observed that they induce a smaller increase in area/lipid. This may be explained by their preferential location at the membrane/water interface and by their mostly vertical orientation on the lipid membrane. In turn, the seven molecules from Group 2 were responsible for a larger membrane expansion, which is in accordance with the previously presented data on the formation of membrane defects (Figure 3). 

#### 3.1.4. Hydrogen Bonding

**Figure 7:**
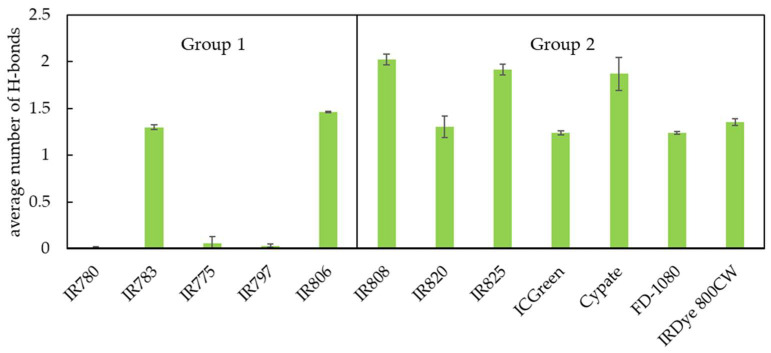
Analysis of the average number of hydrogen bonds established between the different NIR probes inserted in the membrane and water molecules.

Considering that membrane defects drive water molecules into the membrane, we also analyzed the formation of H-bonds between the probes and the water molecules. For that purpose, we calculated the number of hydrogen bonds established between the different NIR probes while inserted in the membrane and the solvent water molecules. This analysis was conducted considering water molecules as donors for the hydrogen bonds for the oxygen (O) and nitrogen (N) acceptor atoms in the structure of the NIR molecules. Moreover, the number of hydrogen bonds was normalized taking into account the different number of acceptor atoms present in the different NIR molecules. The results are shown in Figure 7. For the molecules of group 1, IR783 and IR806 with charged R1 and R2 groups establish the higher number of H-bonds due to the presence of O atoms. For IR780, IR775, and IR797, the sole presence of N acceptor atoms and the good packing of these molecules in the lipid membrane significantly reduce the number of H-bonds that are established. This may also be related to the deeper location induced by the heptamethine bridge in the membranes [64]. For the molecules of group 2, besides the normalization of the results considering the different number of H-bond acceptor groups, and the fact that some molecules stay deeper in the membrane, because these molecules induce a larger perturbation, they also establish a higher number of H-bonds with water. Notably, the number of H-bonds of the molecules from group 2 does not decrease considerably when compared to the results for other molecules that locate preferentially at the lipid/water interface, due to their ability to induce water defects deeply in the membrane.

### 3.2. Experimental Characterization

**Figure 8:**
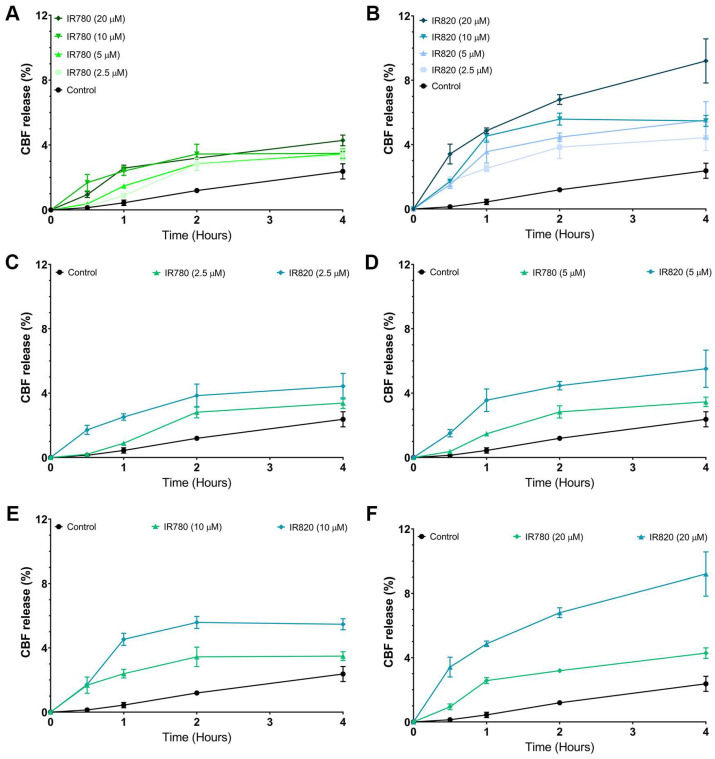
Effect of IR780 and IR820 on the release of CBF trapped in lipid vesicles. Plots (**A**,**B**) show the time dependence of the release with different concentrations of IR780 and IR820, respectively. Plots (**C**–**F**) show the time dependence of the release for IR780 and IR820 at the incubation concentrations of 2.5, 5, 10, and 20 μM.

The results from the MD simulations led to the classification of the NIR molecules addressed in this study into two separate groups, due to their location and behavior in the lipid membrane, but mainly due to their ability to induce perturbations on the structure of the lipid membranes, which are expected to translate into distinct experimental effects on lipid membranes. Therefore, the simulation results predicted that the molecules from group 1 induce no or mild perturbations on the lipid membranes, while the molecules from group 2 will induce larger perturbations on lipid membranes. To test this hypothesis and simultaneously validate the simulations’ results, we carried out membrane leakage experiments. For that purpose, a fluorescent probe CBF was initially trapped on lipid vesicles, and the CBF release profile was analyzed upon incubation with one molecule from each group in the study. The resulting CBF@RBC-membrane-derived vesicles had a mean diameter of 191.1 ± 5.5 nm (polydispersity index of 0.22) and zeta potential of −25.2 ± 1.8 mV (Figure S3). Moreover, the CBF loading reached 202 µg of CBF per mg of RBC-membrane-derived vesicles and an encapsulation efficiency of 17%. These values are in accordance with the data available in the literature for RBC membrane-derived vesicles prepared with a similar methodology [71,72,73]. As shown in Figure 8, the membrane leakage in the presence of IR780 and IR820 was followed over time and for different concentrations of each NIR molecule. 

The experimental data from Figure 8 were used to validate the simulation results. The release profiles for different concentrations of IR780 (Figure 8A) indicate that, although the % of CBF release increases with the concentration of IR780, the release at 4 h is similar for all concentrations used, even for 20 mM. Such data indicate that the possible membrane perturbations induced by IR780 should be small. On the other hand, the vesicles exposed to different concentrations of IR820 (Figure 8B) showed an increase in the CBF release, with more evidence for the release profile at 20 mM. The comparison of Figure 8A,B for IR780 and IR820, respectively, indicates that IR820, included in the molecules of group 2 by the MD simulations, induces larger perturbations of the lipid membranes; additionally, this effect should be more pronounced with the increase in the IR820 concentration, leading to a higher % of CBF release. From the analysis of Figure 8C–F, it can be observed that the molecule of group 2 (IR820) is more efficient in inducing membrane leakage for all tested concentrations. Although it can be a quite unexpected result due to the lower hydrophobicity of probes containing R1 and R2 charged groups, such as IR820, this agrees with the ability of the molecules from group 2 to be deeply inserted in the lipid membrane and create membrane defects that ultimately may lead to the disruption of the membrane. Indeed, the data available in the literature point to a higher cytotoxicity of IR820 in MCF-7 cells in the absence of irradiation, i.e., cell viability of 75% for IR820 at 8 µM [74], while IR780 at 7.5 µM results in a cell viability higher than 80% [75]. Additionally, in another work, the authors showed a small increase in the BMS202 release without NIR laser irradiation when the IR780 was also loaded on lipid nanoparticles—a total release rate of 56.7% and 51.4% at 72 h in the presence and absence of IR780, respectively [76].

As referred to in the methods section, the MD simulations were carried out in lipid bilayers composed of POPC. On the other hand, in the leakage assays, the lipid extract from red blood cells was used due to their closer proximity with a physiological membrane model. We anticipate that, if differences would arise due to the use of two different lipid compositions, it would be reflected in a higher resistance to deformations by the red blood cell extracts, both due to their lipid diversity and due to the presence of cholesterol. This effect would have been translated into an increased difficulty for the validation of the MD simulations hypothesis, and not the opposite direction. In the future, MD simulations of those NIR probes should be conducted in lipid bilayers with more complex and realistic lipid compositions. Additionally, simulations with different numbers of probes can also be used to extract additional features from the membrane leakage process [58,77]. In addition, these simulations can be conjugated with results from other biophysical tools appropriate for confirming the extent of the probe disruption of structural, nanomechanical, and thermal properties [77,78,79,80].

## 4. Conclusions

This work aims to contribute to the establishment of structure–activity relationships for the use of the interaction and behavior of NIR probes with lipid bilayers and also with cell membranes. The description of the role of the NIR probes’ structural features in their diverse applications is of utmost importance for the rational selection of one among the many NIR probes available for a given application, such as cancer therapy. For this, we combined MD simulations with membrane leakage assays to assess one of the experimental applications of these probes, the disruption of cell membranes. 

The MD simulations allowed for a detailed characterization of the interaction of the NIR probes with cell membrane models. From the MD results, the tested molecules were divided into two groups considering their behavior while inserted in the lipid membranes, including their ability to induce membrane defects. From the different molecules, those with neutral R1 and R2 substituents, placed in group 1, did not show a significant capacity for perturbing the lipid bilayer. Molecules with charged R1 and R2, which function as two polar anchors for the aqueous phase, were placed in group 2, being more prone to perturb the structure of lipid membranes by the formation of defects and pores. The grouping of molecules carried out in this work is not strictly based on the nature of their R1 and R2 substituents, since IR783 was attributed to group 1. In this case, the combination between the length of the R1 and R2 substituents with the ring structure of the probe predicts a preferential location for all molecules of this probe at the membrane/water interface. 

Finally, the simulation results were experimentally validated. As predicted, the molecules from group 2 induce a higher leakage efficiency than the molecules from group 1. This is related to the higher ability of molecules from group 2 to induce membrane defects and pores. From the results reported in this work, we may extrapolate that the observed membrane defects and pores should occur during the translocation process of these molecules, which can be hypothesized to be accelerated during NIR light irradiation. Further biophysical characterization of the interaction of the NIR probes with lipid membranes would be very useful for a deeper understanding of the behavior of these highly promising molecules with the application in PTT, PDT, and bioimaging. Therefore, more important than the probe categorization used in this work is the general knowledge about the possible mechanisms behind the biological activity of these molecules. In this context, the perturbation of the lipid bilayers is just a piece in the puzzle. It is not possible to discard other specific behaviors of these molecules in more specific lipid bilayers. Additionally, the importance of other activity mechanisms induced by the irradiation of these molecules cannot be ruled out, namely, through the production of reactive oxygen species whose activity will add to the intrinsic activity of each molecule. 

## Data Availability

All necessary input files may be obtained upon request.

## Supplementary Materials

The following supporting information can be downloaded at: https://www.mdpi.com/article/10.3390/pharmaceutics15071853/s1, Figure S1—Density Maps of the water molecules for the representative molecular dynamics simulations for each of the NIR probes included in Group 1. Density values, shown in color scale (see scale bar), are averaged along the x direction, for given y and z. Figure S2—Density Maps of the water molecules for the representative molecular dynamics simulations for each of the NIR probes included in Group 2. Density values, shown in color scale (see scale bar), are averaged along the x direction, for given y and z. Figure S3—Analysis of the size and charge of CBF@RBC-membrane-derived vesicles. Plot (A) show the size distribution of CBF@RBC-membrane-derived vesicles obtained through the dynamic light scattering analysis. Plots (B), (C), and (D) show the mean size, polydispersity index (PDI), and zeta potential of CBF@RBC-membrane-derived vesicles, respectively.
