# Supplementary material for: Interaction of Near-Infrared (NIR)-Light Responsive Probes with Lipid Membranes: A Combined Simulation and Experimental Study
Source: Pharmaceutics. 2023 Jun 30;15(7):1853. doi: 10.3390/pharmaceutics15071853 (PMC10383845; doi:10.3390/pharmaceutics15071853)
Supplement: Supplementary file 1 [file pharmaceutics-15-01853-s001.zip › pharmaceutics-2441441-supplementary.pdf]

Supplemental Information for

Interaction of near infrared (NIR)-light responsive probes with lipid membranes: a combined simulation and experimental study

Hugo A. L. Filipe [1,2,*], André F. Moreira [1,3,*], Sónia P. Miguel [1,3], Maximiano P. Ribeiro [1,3] and Paula Coutinho [1,3]

1 CPIRN-IPG—Center of Potential and Innovation of Natural Resources, Polytechnic Institute of Guarda, 6300-559 Guarda, Portugal; spmiguel@ipg.pt (S.P.M.); mribeiro@ipg.pt (M.P.R.); coutinho@ipg.pt (P.C.)

2 Coimbra Chemistry Center—Institute of Molecular Sciences (CQC-IMS), University of Coimbra, 3004-535 Coimbra, Portugal

3 CICS-UBI—Centro de Investigação em Ciências da Saúde, Universidade da Beira Interior, 6200-506 Covilhã, Portugal

* Correspondence: hlfilipe@ipg.pt (H.A.L.F.); afmoreira@ipg.pt (A.F.M.)

Contents

1. **Density Maps of the MD simulation systems** **2**
2. **Characterization of CBF@RBC-membrane-derived vesicles** **4**

1. Density Maps of the MD simulation systems

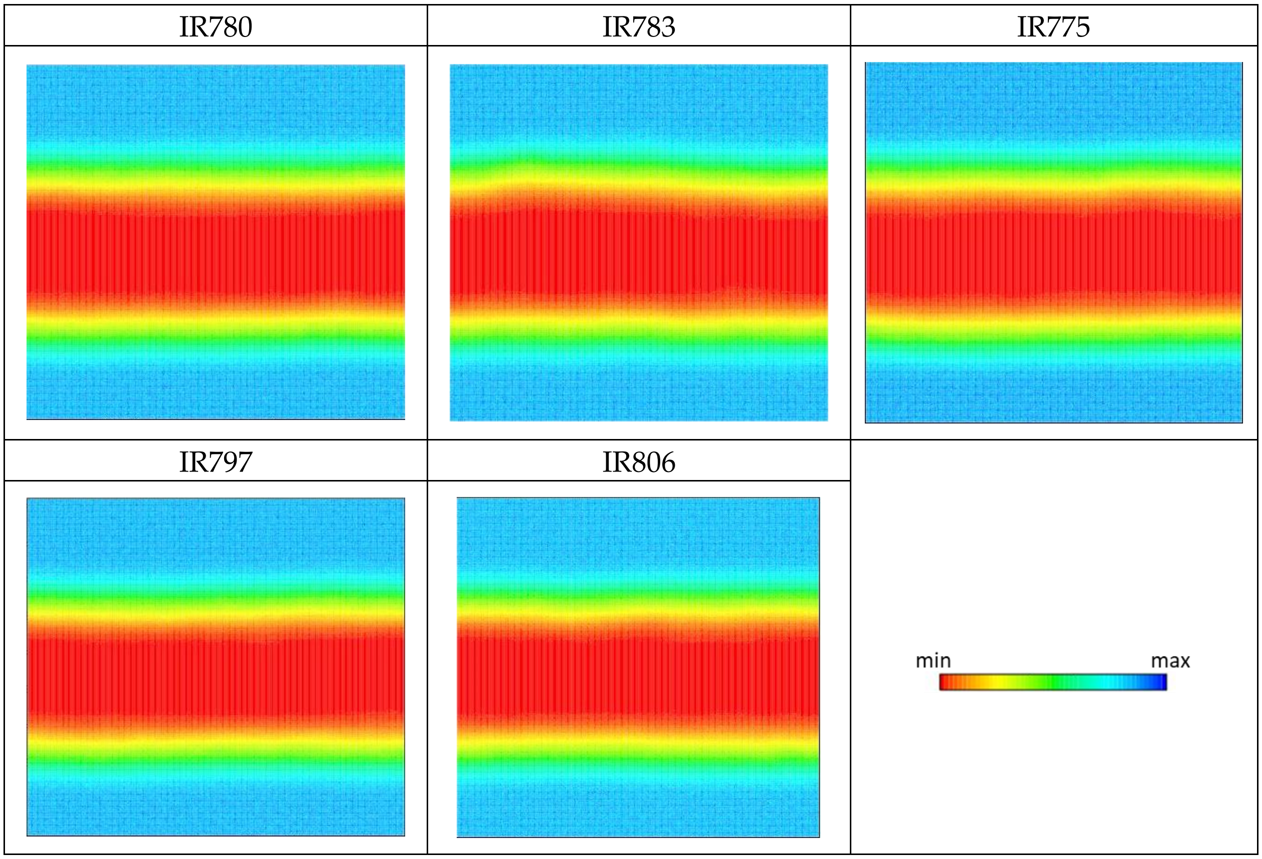

Figure S1 – Density Maps of the water molecules for the representative molecular dynamics simulations for each of the NIR probes included in Group 1. Density values, shown in color scale (see scale bar), are averaged along the x direction, for given y and z.

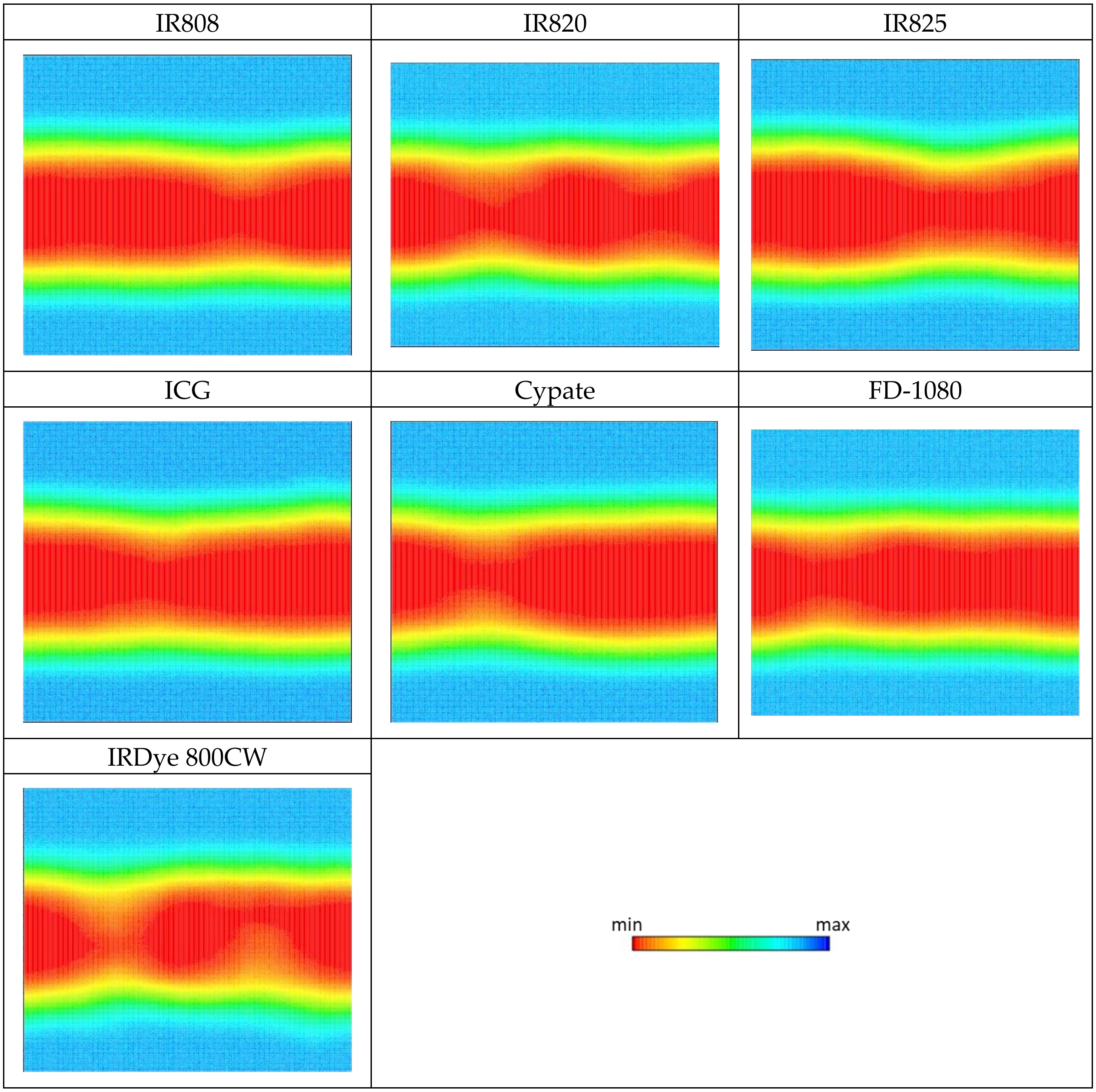

Figure S2 – Density Maps of the water molecules for the representative molecular dynamics simulations for each of the NIR probes included in Group 2. Density values, shown in color scale (see scale bar), are averaged along the x direction, for given y and z.

2. Characterization of CBF@RBC-membrane-derived vesicles

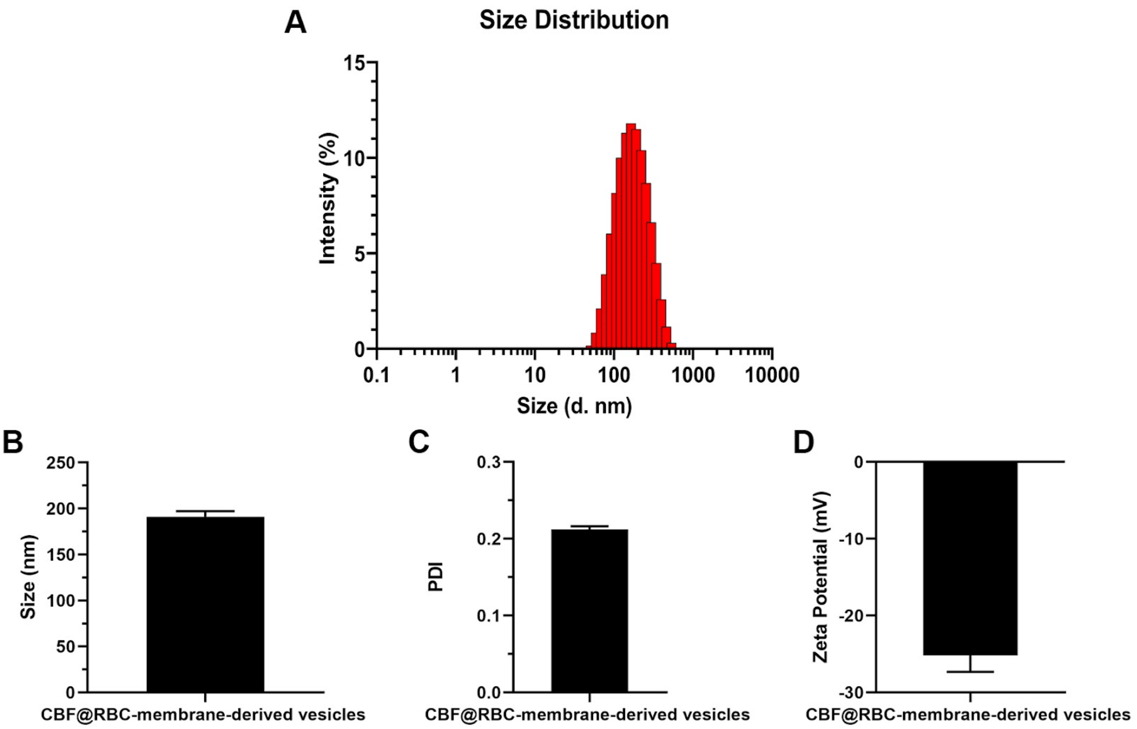

Figure S3 – Analysis of the size and charge of CBF@RBC-membrane-derived vesicles. Plot A) show the size distribution of CBF@RBC-membrane-derived vesicles obtained through the dynamic light scattering analysis. Plots B), C), and D) show the mean size, polydispersity index (PDI), and zeta potential of CBF@RBC-membrane-derived vesicles, respectively.
